# Assessment of Various Tissues in Broilers Reared Under Different Lighting Programs with Respect to Rearing Disorders

**DOI:** 10.3390/vetsci13010075

**Published:** 2026-01-12

**Authors:** Umut Can Gündoğar, Ozan Ahlat, Esin Ebru Onbaşılar

**Affiliations:** 1 Department of Animal Husbandry, Faculty of Veterinary Medicine, Ankara University, 06070 Ankara, Türkiye; ucgundogar@ankara.edu.tr (U.C.G.); onbasilar@ankara.edu.tr (E.E.O.); 2 Department of Pathology, Faculty of Veterinary Medicine, Ankara University, 06070 Ankara, Türkiye

**Keywords:** broiler, histomorphometry, histopathology, lighting, rearing disorders

## Abstract

This study aimed to assess the impact of gradual versus abrupt light–dark transitions on the pectoral muscle, heart, tibia, and eye tissues of broilers, focusing on rearing disorders. A total of 270 male broiler chicks (ROSS-308) were divided into three groups according to the type of light transition: abrupt, 30-min gradual, and 1-h gradual changes in light intensity. The broilers were reared for six weeks, after which samples were collected for gross examination, morphometric and histomorphometric measurements, and histopathological analysis of the pectoral muscle, heart, tibia, and eye. White striping incidence in the pectoral muscle was not significantly affected by light–dark transition type. Results indicated that broilers in the 30-min gradual transition group had a higher relative heart weight than those in the abrupt-transition group (*p* < 0.05), although the RV/TV ratio did not differ. Tibial measurements, including weight, length, and cortical index, showed no differences across groups. Broilers exposed to a 1-h gradual transition between light and dark periods showed significantly greater eye weights compared to both the birds in the abrupt transition group and the birds in the 30-min transition group (*p* < 0.01). Overall, gradual light transitions did not substantially affect pectoral muscle or bone health but had minor effects on heart and eye weights in broilers reared under intensive production systems.

## Simple Summary

Lighting programs are an important management factor in intensive broiler production. This study evaluated whether abrupt or gradual transitions between light and dark periods influence organ development and the occurrence of rearing-related disorders in broilers. The findings showed that different light transition regimes did not affect the occurrence of major musculoskeletal disorders. Gradual light transitions resulted in minor differences in some organ weights but did not lead to clear health advantages compared to abrupt transitions. These results indicate that extending light–dark transition periods beyond short gradual changes may not provide additional practical benefits in broiler production.

## 1. Introduction

The increasing global population in dietary preferences have led to a growing demand for high-quality animal-derived protein [1]. However, conventional meat production systems used to meet this demand present significant challenges in terms of animal welfare and sustainability [2]. Consequently, modern broiler production systems are required to adopt strategies that not only maximize productivity but also safeguard animal health and welfare. Intensive genetic selection for rapid growth in broilers has enhanced feed conversion efficiency [3,4,5] and increased meat yield, particularly breast muscle proportion [6], but has simultaneously elevated protein synthesis rates and oxygen demand [7]. As the global demand for high-quality broiler meat continues to rise, achieving an optimal balance between productivity and animal welfare has become increasingly critical. In this context, advancements in broiler rearing systems aim to integrate productivity, welfare, and sustainability; however, a thorough understanding of how specific environmental factors influence broiler performance remains essential. Among these factors, light management plays a pivotal role, as it directly affects growth, behavior, health, and overall welfare in broilers [8,9].

Lighting programs, including light source, intensity, photoperiod, and wavelength, significantly impact broiler performance [10,11,12,13,14]. Broilers are typically exposed to various lighting regimes, including continuous, intermittent, and fixed lighting, as regulated by the EU Commission Directive (2007/43/EU), which mandates a minimum of six hours of darkness per day, including at least four consecutive hours. However, the effects of gradual versus abrupt light transitions on broiler health and welfare remain inadequately explored. In natural environments, transitions between day and night occur gradually, whereas intensive broiler production systems often implement abrupt light–dark cycles. Previous studies [14] suggest that gradual transitions may promote calmness, enhance immune function, and reduce stress, potentially alleviating the risk of rearing disorders.

In broiler production, key tissues such as the pectoral muscle, heart, tibia, and eyes are integral to ensuring optimal growth, welfare, and overall performance. The pectoral muscle, a primary component of poultry meat production, directly impacts meat yield and quality [6], and optimal muscle development is essential for both growth efficiency and economic performance. The heart is essential for supplying oxygen and nutrients to support the rapid growth of broilers, particularly in intensive systems where growth rates are elevated [7]. When heart development fails to keep pace with increases in body weight, often due to environmental or nutritional stressors, conditions such as ascites and right ventricular hypertrophy may arise [15]. The tibia serves as a primary skeletal support, critical for maintaining proper locomotion during rapid growth [16]. Tibial dyschondroplasia (TD), characterized by avascular cartilage of the tibia, represents a significant challenge for intensive production systems [17]. Lastly, eye health in broilers is influenced by light–dark cycles, affecting physiological processes such as growth, vision, and general well-being [18]. Disruptions to these cycles, especially those caused by abrupt light transitions, may pose risks to ocular health and performance [19].

Collectively, these organs are fundamental to ensuring the sustainability and profitability of broiler production systems, necessitating management practices that optimize both production efficiency and animal welfare. This study aims to investigate the impact of different light transition programs on the pectoral muscles, heart, tibia, and eye tissues of broilers, focusing on their potential effects on rearing disorders such as white striping, ascites, tibial dyschondroplasia, and ocular abnormalities. We hypothesize that gradual light–dark transitions will reduce rearing disorders, offering a potential pathway to improving broiler health in intensive production systems.

## 2. Materials and Methods

### 2.1. Animals and Experimental Design

In the study, 270 day-old male broiler chicks (ROSS-308) were reared under the different lighting regimes that varied according to whether the transitions between light and dark periods during the day were abrupt or gradual (by changing light intensity). The sample size was determined based on a power analysis assuming a moderate-to-large effect size (Cohen’s f = 0.40), α = 0.05 and power = 0.80. The analysis was performed using G*Power software (version 3.1). Male broilers are known to achieve higher body weights during the fattening period and are more prone to rearing disorders. Therefore, the use of male chicks allowed a more controlled evaluation of the effects of lighting transition regimes on related traits. Daylight-colored LED bulbs were used in all groups. The abrupt transition group does not include a familiarization period between light and dark periods during the day. In the second group, light intensity between light–dark and dark–light periods during the day was gradually reduced within 30 min using a dimmer and increased within 30 min. In the 3rd group, light intensity was gradually reduced within 1 h and increased within 1 h. For this purpose, the broiler house was divided into three rooms to prevent light transmission. In each room, 10 compartments measuring 90 × 80 cm were created. Nine chicks were placed in each compartment. Each group consisted of 10 repetitions. For the first 3 weeks of the study, chicks were fed with feed containing 23.23% CP and 3010 kcal/kg ME and then they were fed with feed containing 19.63% CP and 3196 kcal/kg ME. During days 0–7 of the experiment, plastic chick feeders and chick drinkers were used, and from day 8 to 42, hanging feeders and nipple drinkers were used. After providing a temperature of 32 ± 1 °C at the chick level for the first three days, the temperature was gradually reduced until the age of 21 days and then maintained at a constant 22 ± 1 °C [20]. At the six-week-old, 10 broilers were chosen from each group (a total of 30 broilers from all groups) and they were weighted and slaughtered [14]. All broilers within each pen were weighed individually using a precision digital scale (TSC-30, Necklife, Shenzhen, China). Since each treatment group consisted of 10 replicates (pens), ten broilers were slaughtered per group to ensure that each replicate was represented. Accordingly, one broiler was selected from each pen to allow a balanced reflection of within-group variation. To minimize potential selection bias, the broilers selected from each pen were those whose final body weights were closest to the respective group means at the end of the experimental period. Broilers were slaughtered by severing the jugular veins and carotid arteries in accordance with standard poultry slaughtering procedures. The pectoral muscle (M. pectoralis major), heart, tibia and eyes were removed for further analyses.

### 2.2. Gross Examination and Morphometric Measurements

The pectoral muscles of all slaughtered broilers were removed from the carcass and examined for the presence of white striping. White striping in the pectoral muscle was macroscopically evaluated based on the criteria outlined by Kuttappan et al. [21]. Score 0 represented no visible white stripes on the surface, Score 1 indicated thin white stripes (<1 mm), and Score 2 denoted prominent white stripes (>1 mm) across the surface. Then muscle samples were collected from lesional area of the pectoral muscle. Macroscopical evaluations were performed by an experienced investigator using a blinded approach, independent of the treatment groups. Prior to scoring, the evaluation criteria were standardized and calibration was conducted using reference samples. This procedure minimized observer-related bias and ensured consistency in the assessments.

After determining heart weight, relative heart weight was calculated as the ratio of heart weight to live body weight. The atrium, major vessels, and adipose tissue were removed from the heart, and the remaining lower section was vertically divided to obtain the weights of the right ventricle (RV) and total ventricle (TV), enabling the calculation of the RV/TV ratio [22].

After separation, the tibia from slaughtered birds were cleaned of surrounding muscles. Tibia weights were measured to a precision scale (AJ-3200CE, Vibra, Tokyo, Japan) and relative bone weights were calculated as a proportion of live body weight. For cortical index calculations, tibia was horizontally cut at the midpoint. The diaphysis diameter and medullary canal diameter of the tibia were measured with a digital caliper [23]. Using these measurements, the cortical index was calculated as [(Diaphysis diameter–Medullary canal diameter)/Diaphysis diameter] × 100, and the bone strength index as (Bone length/3√Bone weight) × 100 [24,25]. For grading tibial dyschondroplasia, the proximal part of the left tibia was vertically divided. In examining the proximal metaphysis, the severity of tibial dyschondroplasia was graded based on the extent of the lesion: 1 (no lesion), 2 (lesion spread < 0.5 cm), 3 (lesion spread 0.5–1 cm), and 4 (lesion spread > 1 cm) [26].

Eye weights were recorded after removing the eyes from all slaughtered birds, and relative eye weights were calculated by dividing by the slaughter weight. Eye dimensions including corneal diameter, mediolateral diameter, dorsoventral diameter, and anteroposterior diameter were measured. Eye dimensions were measured with a digital caliper. The length of the corneal diameter was measured as the distance from one edge of the cornea to the other along the vertical axis; the mediolateral diameter was measured as the horizontal distance from side to side at the widest point of the eye; the dorsoventral diameter was measured as the vertical distance at the widest point of the eye and the anteroposterior diameter was measured as the distance from the front of the cornea to the back of the retina [27,28].

### 2.3. Histopathological Examination

The pectoral muscle, heart (including the right ventricular free wall and the interventricular septum) and eye tissues were fixed in 10% neutral buffered formalin for 24–48 h, trimmed, and washed in running tap water. Samples were processed through graded alcohol and xylene series in a tissue processor (Epredia STP 120, Shandon Diagnostics Ltd., Runcorn, UK) and embedded in paraffin (Epredia HistoStar, Shandon Diagnostics Ltd., Runcorn, UK). Five µm sections were cut from the paraffin blocks by rotary microtome (Thermo Scientific HM 355S, Thermo Shandon Ltd., Runcorn, UK), stained with routine hematoxylin and eosin (HE) and mounted with entellan using an automatic stainer and coverslipper (Leica Autostainer XL ST5010-CV5030, Nussloch, Germany). The prepared slides were examined under a light microscope equipped with a camera (Olympus BX51-DP71, Tokyo, Japan). The histopathological findings such as degeneration, necrosis, regeneration, fibrosis, adipose tissue infiltration, and mononuclear cell infiltration in the pectoral muscle were graded semiquantitatively as follows: 0: normal; 1: moderate; 2: severe.

### 2.4. Histomorphometric Analyses of the Eyes

**Figure 1:**
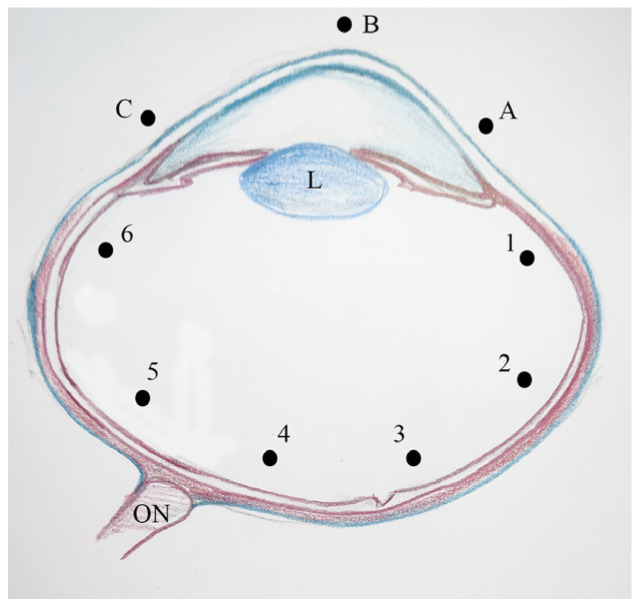
Representative schematic diagram illustrating the measurement points on the cornea (A–C) and retina (1–6), L: Lens, ON: Optic nerve.

Corneal thickness was measured at three different points (A, B, and C), while retinal thickness was measured at six points (1–6) at the fundus [29]. Measurement points were defined on vertically (dorsoventrally) sectioned eyes in accordance with published data. These points are also shown in Figure 1. Histomorphometric analyses of the eyes were performed using the Olympus CellSens software (CS-ST-V1.8).

### 2.5. Statistical Analyses

The normal distribution suitability of the data was examined with the Kolmogorov–Smirnov test. One-way analysis of variance (ANOVA) was used for calculations and to determine differences in mean values among groups for data showing normal distribution. Tukey’s test was applied to determine the group causing the difference. The differences in microscopic characteristics in the muscle samples among storage conditions were analyzed using the Kruskal–Wallis test for independent samples, with a significance threshold set at *p* < 0.05.

White striping score and tibial dyschondroplasia score were recorded as ordinal categorical variables and summarized as frequencies. Comparisons of score distributions among the three lighting transition groups were performed using Pearson’s chi-square test. Given the ordinal nature of the scores, linear-by-linear association tests were additionally used to assess potential trend effects across groups. Effect sizes were quantified using Cramér’s V. A two-sided *p* value < 0.05 was considered statistically significant.

## 3. Results

The incidence of white striping scores among the groups demonstrated variability (Table 1). In the abrupt transition group, 30% of samples scored 0 (no stripes), while the 30-min transition group had 10%, and the 1-h transition group showed a higher incidence at 40%. The differences in Score 0 did not reach statistical significance (X^2^ = 2.39, *p* > 0.05).

The distribution of white striping scores did not differ significantly among lighting transition groups (Pearson’s χ^2^ = 2.83, *p* = 0.586). The effect size was small to moderate (Cramér’s V = 0.22). No significant linear trend across groups was detected (linear-by-linear association, *p* > 0.05).

**Figure 2:**
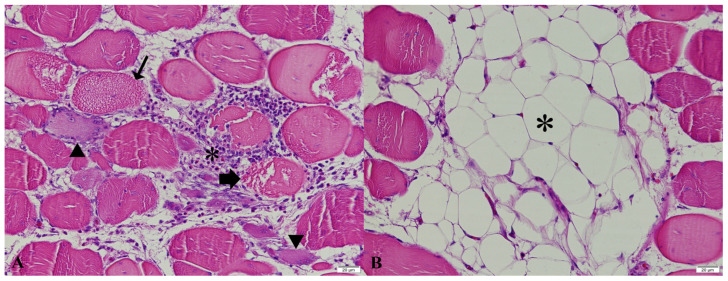
Histopathological findings in pectoral muscle, HE, 1-h transition. (**A**) Degeneration (thin arrow) and necrosis (thick arrow) in muscle fibers, with mononuclear cell infiltration (star) in the interstitial area along with regenerative myocytes (arrowhead). (**B**) Adipose tissue infiltration (star) between muscle fibers.

Histopathological examination of the pectoral muscles exhibiting macroscopic white striping revealed varying degrees of floccular/vacuolar degeneration, necrosis, regeneration (nuclear rowing and multinucleated cells), fibrosis, adipose tissue infiltration, and mononuclear cell infiltration (Figure 2), with no statistically significant differences observed among the groups (*p* > 0.05, Table 2).

The relative heart weight differed significantly among the experimental groups (Table 3). Broilers reared under abrupt light–dark transitions exhibited a significantly lower relative heart weight compared to those in the 30-min transition group, whereas no significant difference was observed between the abrupt and 1-h transition groups. The RV/TV ratio remained consistent across all groups, with values of 0.21 for abrupt transition, 0.22 for 30-min transition, and 0.22 for 1-h transition, indicating no statistical significance (*p* > 0.05).

**Figure 3:**
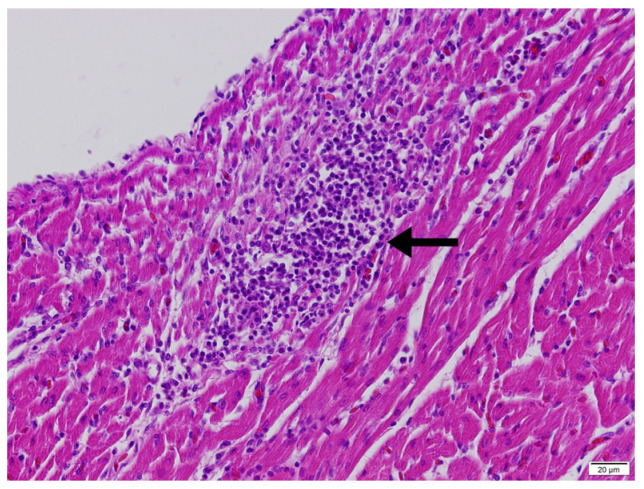
Mononuclear cell infiltration (arrow) in the myocardium, right ventricular free wall, heart, HE, abrupt transition.

Across all groups, the ventricular cardiomyocytes of the right ventricular free wall and interventricular septum generally exhibited a loss of their striated structure and instead showed a granular and/or homogeneously eosinophilic appearance. Mononuclear cell infiltrations, predominantly composed of lymphocytes and macrophages, were observed in the epicardium and myocardium of the right ventricular free wall (Figure 3). In some cases, these infiltrations were accompanied by heterophils and/or eosinophils.

None of the tibia characteristics evaluated—including weight, length, diameter, cortical index, and strength index—were significantly affected by the different light transition regimes (*p* > 0.05; Table 4).

The distribution of tibial dyschondroplasia scores (Table 5) did not differ among lighting transition groups (Pearson’s χ^2^ = 0.67, *p* = 0.95), with a small effect size (Cramér’s V = 0.10). No significant linear trend across groups was observed (linear-by-linear association, *p* > 0.05).

Eye weight and relative eye weight differed significantly among the experimental groups (Table 6). Both the abrupt and 30-min light–dark transition groups exhibited significantly lower eye weight and relative eye weight compared to the 1-h transition group (*p* < 0.01), while no significant differences were observed between the abrupt and 30-min transition groups. Other ocular parameters, including corneal diameter and ocular diameters, were not significantly influenced by the lighting treatments.

**Figure 4:**
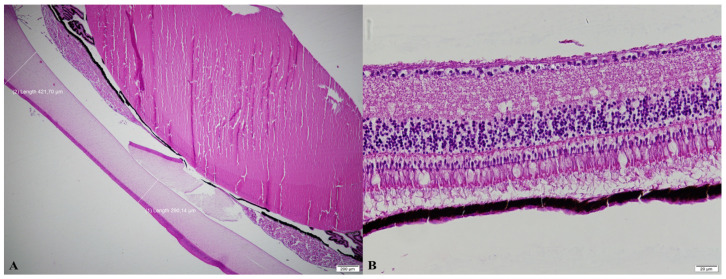
Histomorphometry of the corneal (**A**) and retinal (**B**) thickness, HE, (**A**) abrupt transition, (**B**) 1-h transition.

No significant differences were detected in corneal or retinal thickness measurements at any of the evaluated points among the experimental groups (*p* > 0.05; Table 7). Representative histological sections illustrating corneal and retinal thickness measurements in the abrupt and 1-h light–dark transition groups are presented in Figure 4.

## 4. Discussion

White striping is a myopathy characterized by the presence of white striations in the breast muscle and has become increasingly prevalent in broilers, particularly in heavier birds [21]. Its development is influenced by both genetic predisposition and rearing conditions that promote rapid growth. Bailey et al. [30] reported that although the heritability of white striping is relatively low, environmental factors play a substantial role in its occurrence. This condition primarily affects the pectoral muscles and is closely associated with the intensive practices of modern poultry production systems [31]. Histopathologically, white striping is characterized by degenerative myopathic lesions accompanied by mild regenerative changes, with muscle fibers partially replaced by adipocytes and fibrotic tissue [21]. Our histopathological findings were consistent with these established characteristics; however, no treatment-related differences were detected among the lighting transition groups. This suggests that altering the duration of light–dark transitions alone is insufficient to modulate the development of white striping under intensive rearing conditions.

In fast-growing broilers, heart development often lags behind the rapid increase in body weight, which can lead to serious conditions such as ascites. Ascites in broilers is typically associated with high growth rates and environmental stressors, and it results from right ventricular failure, following dilation and hypertrophy induced by pulmonary hypertension [15]. This condition is primarily driven by the rapid growth of birds that possess limited pulmonary vascular capacity, prompting the right ventricle to hypertrophy in an effort to accommodate the increased workload [32]. In this study, broilers in the abrupt light–dark transition group exhibited a significantly lower relative heart weight compared to those in the 30-min transition group; however, there is no strong evidence indicating that this difference reflects a clinically or physiologically meaningful cardiac disorder. The right ventricle to total ventricle (RV/TV) ratio, which is considered an important indicator of ascites risk, was similar across all groups and remained well below the threshold value of 0.28 associated with ascites development [15]. These findings indicate that the observed difference in relative heart weight is not associated with ascites development or overt heart failure. The presence of only minimal degenerative and inflammatory changes in the myocardium, together with the absence of systemic infectious pathology in other tissues, suggests that these alterations are more likely indicative of a stress-related physiological adaptation to abrupt light transitions rather than a pathological process.

Leg weakness, a common issue in intensive broiler production, is associated with significant economic losses [33]. The rapid growth of broiler long bones, particularly the tibia, involves elongation through chondrocyte proliferation and differentiation in the epiphyseal growth plates [16]. Several factors during rearing, including poor litter, and environmental stressors such as high temperatures, humidity, and overcrowding, can contribute to leg disorders. Studies typically focus on light duration, intensity, and color. Yang et al. [34] observed leg abnormalities across three experimental treatments; however, the bone elastic modulus of birds exposed to intermittent light (4 L:4 D) was higher than that of birds under the intermittent light (2 L:2 D) and continuous light regimes. The endogenous melatonin rhythm plays a role in regulating key markers involved in bone physiology [35]. Research on broilers and turkeys with varying lighting programs indicated that longer lighting durations increased mortality, reduced mobility, and altered behavior in both species [36]. High-intensity lighting has been shown to support tibial growth and mitigate leg diseases by promoting weight gain aligned with skeletal development. In this study, however, no significant differences were observed in tibial morphometric characteristics across the different light transition groups, suggesting that light transitions did not influence tibial health or development. Additionally, the incidence of tibial dyschondroplasia, a condition characterized by unvascularized cartilage in the tibia [17], did not differ significantly among the groups, further supporting the conclusion that light transition periods did not impact leg health. This suggests that other environmental or nutritional factors may play a more dominant role in the development of leg disorders than light transition characteristics alone.

Eye growth in birds is regulated by light–dark cycles, with increased growth occurring during the light phase and reduced growth during the dark phase, influenced by hormones like dopamine and melatonin [18,37]. Disruptions to this cycle can lead to ocular abnormalities. Studies have shown that prolonged daylength or low light intensity can alter eye dimensions and potentially increase the risk of ocular diseases [19,38]. In our study, broilers exposed to a 1-h gradual transition between light and dark periods showed significantly greater eye weights compared to both the birds in the abrupt transition group and the birds in the 30-min transition group. This may reflect the longer effective exposure to light during gradual transitions, even at reduced light intensities. However, despite differences in eye weight, no significant changes were detected in corneal or retinal thickness or other ocular morphometric parameters. These findings suggest that while gradual light transitions may influence overall eye growth, they do not appear to induce structural alterations indicative of ocular pathology.

## 5. Conclusions

Overall, the results of this study indicate that modifying the duration of light–dark transitions had limited effects on broiler physiological and histopathological traits. Although gradual light transitions were associated with minor differences in heart weight and eye size, these changes were not accompanied by indicators of impaired cardiac function or pathological alterations. Importantly, light transition regimes did not influence the incidence or severity of pectoral muscle myopathies or skeletal disorders. Taken together, these findings suggest that extending light–dark transition periods does not confer clear health advantages about rearing disorders under intensive broiler production conditions.

## Figures and Tables

**Table 1:** Pectoral muscle white striping scores by group.

Characteristic	Light and Dark Transition Periods			Significance
	Abrupt Transition	30-min Transition	1-h Transition	
White Striping Score *, %				
Score 0	30	10	40	χ^2^ = 2.83, *p* = 0.586, Cramér’s V = 0.22
Score 1	40	60	30	
Score 2	30	30	30	

* Score 0 (no stripes): Pectoral muscle without white striping on the surface; Score 1 (moderate striping): Pectoral muscle with white stripes < 1 mm; Score 2 (severe striping): Pectoral muscle with white stripes > 1 mm across the surface.

**Table 2:** Histopathological examination of pectoral muscles exhibiting macroscopic white striping.

Histopathological Findings	Light and Dark Transition Periods			*p*
	Abrupt Transition	30-min Transition	1-h Transition	
Degeneration	1.0 (0–1)	1.0 (1–1.5)	2.0 (1.0–2.0)	0.064
Necrosis	1.0 (0–1)	1.0 (0.5–1.0)	1.5 (1.0–2.0)	0.101
Regeneration	0 (0–1.0)	0 (0–1)	1.0 (0.0–1.25)	0.481
Fibrosis	0 (0–1.0)	0 (0–0.5)	0.5 (0–1.0)	0.530
Adipose Tissue Infiltration	1.0 (1.0–1.0)	1.0 (1.0–1.5)	1.0 (1.0–2.0)	0.726
Mononuclear Cell Infiltration	1.0 (1.0–1.0)	1.0 (0.5–1.0)	1.0 (1.0–1.0)	0.219

Median values and interquartile ranges (25th–75th percentiles) were reported. Scale used to assess for histopathological changes: 0 = normal, 1 = moderate, 2 = severe.

**Table 3:** Relative heart weight and RV/TV ratio in broilers by group.

Characteristic	Light and Dark Transition Periods			*p*
	Abrupt Transition	30-min Transition	1-h Transition	
Relative heart weight, %	0.53 ± 0.02 ^b^	0.60 ± 0.02 ^a^	0.56 ± 0.01 ^ab^	0.016
RV/TV	0.21 ± 0.02	0.22 ± 0.02	0.22 ± 0.01	0.900

Mean ± Standard Error. a,b: Values with different letters in the same row are statistically significant.

**Table 4:** Tibia characteristics by group.

Characteristic	Light and Dark Transition Periods			*p*
	Abrupt Transition	30-min Transition	1-h Transition	
Tibia weight, g	22.2 ± 0.7	21.5 ± 1.0	22.2 ± 0.6	0.720
Tibia length, mm	111.7 ± 1.3	110.6 ± 1.4	110.9 ± 0.61	0.801
Diaphysis diameter, mm	10.1 ± 0.2	9.8 ± 0.3	10.0 ± 0.2	0.576
Cortical index, mm	29.48 ± 1.08	28.37 ± 0.89	31.44 ± 0.95	0.098
Strength index	3982 ± 37	3990 ± 49	3952 ± 39	0.794

Mean ± Standard Error. *p* > 0.05.

**Table 5:** Incidence of tibial dyschondroplasia by group.

Characteristic	Light and Dark Transition Periods			Significance
	Abrupt Transition	30-min Transition	1-h Transition	
Tibial Dyschondroplasia Score *, %				
Score 1	50	50	50	χ^2^ = 0.67, *p* = 0.95, Cramér’s V = 0.10
Score 2	30	40	40	
Score 3	20	10	10	

* Score 1: No lesion; Score 2: Lesion area < 0.5 cm; Score 3: Lesion area between 0.5 and 1 cm.

**Table 6:** Ocular measurements by group.

Characteristic	Light and Dark Transition Periods			*p*
	Abrupt Transition	30-min Transition	1-h Transition	
Weight, g	2.24 ± 0.07 ^b^	2.20 ± 0.05 ^b^	2.46 ± 0.06 ^a^	0.009
Relative weight, X10^−6^	7.41 ± 0.17 ^b^	7.28 ± 0.23 ^b^	8.27 ± 0.23 ^a^	0.006
Corneal diameter, mm	7.66 ± 0.09	7.80 ± 0.16	8.03 ± 0.10	0.109
Mediolateral diameter, mm	17.36 ± 0.22	17.07 ± 0.23	17.80 ± 0.19	0.071
Dorsoventral diameter, mm	15.99 ± 0.17	16.40 ± 0.25	15.98 ± 0.20	0.280
Anteroposterior diameter, mm	13.04 ± 0.63	13.75 ± 0.20	14.44 ± 0.18	0.061

Mean ± Standard Error. a,b: Values with different letters in the same row are statistically significant.

**Table 7:** Corneal and retinal thickness measurements by group.

Thickness (µm)	Light and Dark Transition Periods			*p*
	Abrupt Transition	30-min Transition	1-h Transition	
Cornea Point A	505.3 ± 240.5	405.2 ± 97.7	436.8 ± 60.0	0.280
Cornea Point B	445.2 ± 117.3	365.1 ± 208.4	355.6 ± 116.1	0.460
Cornea Point C	418.7 ± 78.8	383.5 ± 84.4	452.3 ± 120.6	0.310
Retina Point 1	111.9 ± 25.7	109.2 ± 14.5	110.7 ± 27.3	0.970
Retina Point 2	158.9 ± 46.6	145.3 ± 34.1	157.1 ± 38.7	0.630
Retina Point 3	206.4 ± 45.5	175.7 ± 33.4	192.8 ± 42.4	0.210
Retina Point 4	208.7 ± 44.5	204.5 ± 52.1	192.3 ± 30.3	0.680
Retina Point 5	175.6 ± 19.6	206.0 ± 73.2	185.9 ± 56.2	0.370
Retina Point 6	135.4 ± 21.8	120.6 ± 18.4	113.4 ± 9.2	0.090

Mean ± Standard Error.

## Data Availability

The original contributions presented in this study are included in the article. Further inquiries can be directed to the corresponding author.

## Abbreviations

The following abbreviations are used in this manuscript:

CP	Crude protein
HE	Hematoxylin and eosin
ME	Metabolizable energy
RV	Right ventricle
TD	Tibial dyschondroplasia
TV	Total ventricle
